# Major surgery in an osteosarcoma patient refusing blood transfusion: case report

**DOI:** 10.1186/1477-7819-8-96

**Published:** 2010-11-08

**Authors:** Amreeta Dhanoa, Vivek A Singh, Rukmanikanthan Shanmugam, Raja Rajendram

**Affiliations:** 1 Jeffrey Cheah School of Medicine and Health Sciences, Monash University Sunway Campus, Malaysia; 2 Department of Orthopaedic Surgery, University Malaya Medical Center, Malaysia; 3 Department of Anaesthesia, University Malaya Medical Center, Malaysia

## Abstract

We describe an unusual case of osteosarcoma in a Jehovah's Witness patient who underwent chemotherapy and major surgery without the need for blood transfusion. This 16-year-old girl presented with osteosarcoma of the right proximal tibia requiring proximal tibia resection, followed by endoprosthesis replacement. She was successfully treated with neoadjuvant chemotherapy and surgery with the support of haematinics, granulocyte colony-stimulating factor, recombinant erythropoietin and intraoperative normovolaemic haemodilution. This case illustrates the importance of maintaining effective, open communication and exploring acceptable therapeutic alternative in the management of these patients, whilst still respecting their beliefs.

## Background

Jehovah's Witnesses are well known in the medical world for their refusal on the acceptance of blood and blood products [1]. Unique aspects of these beliefs can pose health care providers with challenging medical, legal and ethical dilemmas. Modifications of standard transfusion practices may be necessary to respect the beliefs of a Jehovah's Witnesses patient and this may be an impediment to optimal care of a patient. We describe here a 16-year-old Jehovah's Witness patient with osteosarcoma who required a major surgery and chemotherapy, which we believe is the first reported such case.

## Case presentation

### Clinical presentation

Miss S is a 16-year-old Chinese girl. She presented to a tertiary hospital with an initial complaint of progressively increasing pain and swelling of her right leg of 3 months duration. It was interfering with her right knee movement and walking. It was not associated with any significant trauma and started insidiously. She did not experience any loss of appetite, loss of weight or fever during and around the time of presentation. She had no other known medical conditions prior to this and was not on any medications.

### Clinical and radiological findings

Examination of the patient showed a medium built girl with a large swelling measuring 10 cm by 15 cm over her right leg, just below the knee. She did not appear wasted and was walking with an antalgic gait. The skin over the swelling appeared shiny, indurated with visible dilated veins overlying it. Her vital signs were normal and there was no evidence of pallor. On palpation, there was a warm hard swelling arising from the proximal right tibia not crossing the knee joint. It was a smooth lobular swelling, tender on deep palpation. Range of motion for the right knee was 0° to 100° compared to 0° to 140° on the contralateral side. There was no clinical evidence of knee effusion. Examination of all other systems was unremarkable.

**Figure 1:**
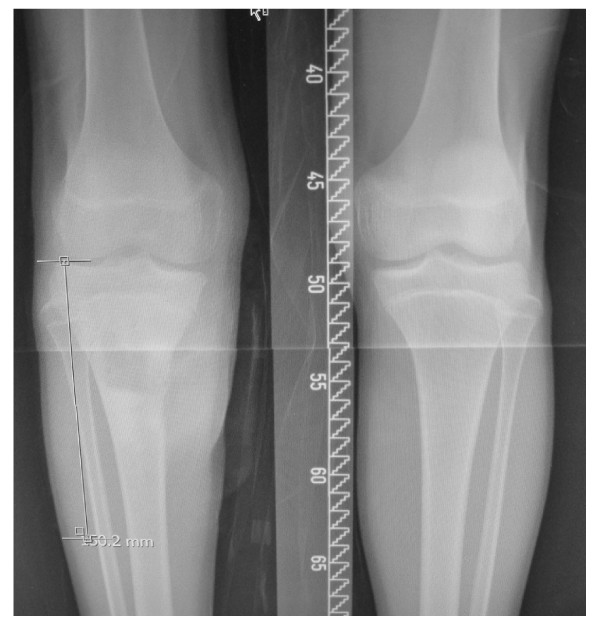
**Plain radiograph showing a mixed sclerosis and lytic lesion over the right upper tibia and break in the medial cortex**.

**Figure 2:**
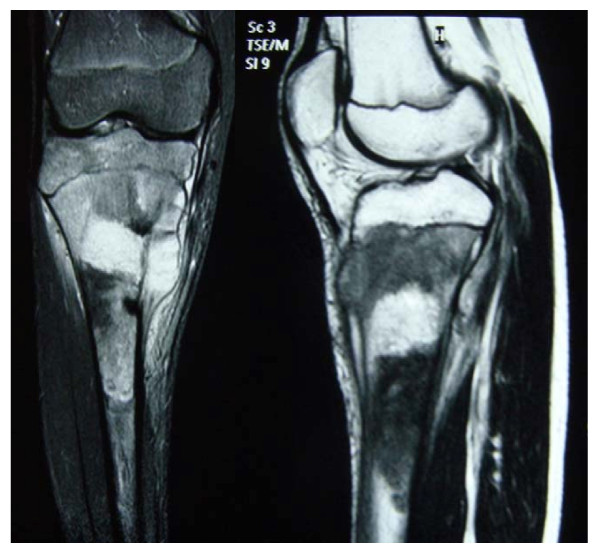
**Magnetic Resonance Scanning of the right tibia showing a tumour within the right upper tibia breaching the medial cortex to extend into the soft tissue medially**.

Plain radiographs (Figure 1) showed classical features consistent with osteosarcoma of the proximal tibia. The Magnetic Resonance Imaging showed that the tumour was limited to the proximal tibia without involvement of the knee joint and the neurovascular bundle was free from the tumour (Figure 2). Computer Tomography of the chest and bone scan revealed that the tumour was localize to right proximal tibia without metastasis to the lung or other bones. The clinical examination and radiological findings were consistent with an initial diagnosis of osteosarcoma of the right proximal tibia. Histopathological findings of a large-core tissue biopsy performed showed chondromyxoid matrix and atypical chondrocytes containing enlarged hyperchromatic nuclei. There were also abnormal spindle cells producing osteoid present. These findings were consistent with chondroblastic variant of osteosarcoma.

### Preoperative Management

During the first encounter with the Orthopaedic Surgeon, the family members confirmed the Jehovah's Witness status of the patient. Subsequently, a meeting between the Orthopaedic Surgeon, the family members and church representatives was held. The Hospital Liaison Committee for Jehovah's Witnesses also sent representatives to provide support to the family and medical literature to the treating doctors for additional information. There was acceptance towards iron and recombinant erythropoietin. However, the family refused packed red blood cells (RBC), whole blood and fresh frozen plasma.

She was started on neoadjuvant chemotherapy which included doxorubicin, cisplatin and high-dose methotrexate with leucovorin (folinic acid) rescue (Memorial Sloan-Kettering protocol). The regime comprised of 6 cycles of chemotherapy. Surgery was performed after 3 cycles of neoadjuvant chemotherapy.

Before commencement of chemotherapy, she was started on ferrous fumarate, folic acid, vitamin B complex and subcutaneous recombinant erythropoietin 50,000 units three times a week. These measures were expected to increase her hemoglobin levels and accelerate red cell production. Immediately after her chemotherapy, she was also given neupogen (granulocyte colony-stimulating factor) to prevent chemotherapy-induced neutropenia. During the course of chemotherapy, her blood counts were stable with the range of recorded hemoglobin of 8.7 to 13.4 g/dL, white blood cell count of 1.9 to 14.8 × 10^9^/L and platelet count of 77 to 268 × 10^9^/L.

Following three cycles of chemotherapy, clinically, there was marked reduction of the tumour mass and patient was prepared for limb salvage surgery. A standard consent for surgery and another one for anaesthesia was obtained from the parents. The parents were clearly informed about the possible risks their child may encounter because of refusal of blood transfusion and this was clearly documented in the medical notes. The patient also had a hand written note describing her religious beliefs and her refusal for blood transfusion, which she showed to all attending doctors. This we believe was because whilst the official medico legal consent form was signed by her parents, she wanted the treating doctors to know that the decision to refuse any form of transfusion was without coercion from external parties.

Three empty blood bags containing anticoagulants routinely used for blood collection were obtained from the blood bank to be used intraoperatively.

### Surgery

**Figure 3:**
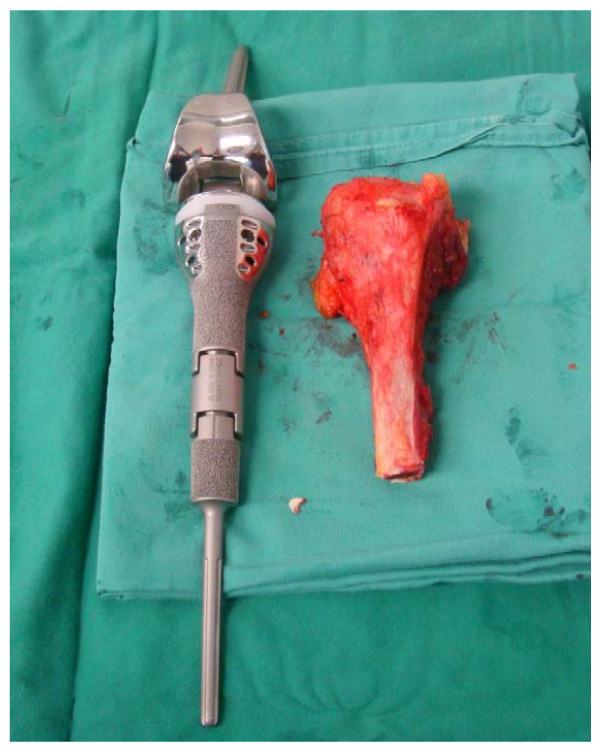
**Resected tibia shown with endoprosthesis used to replace the defect**.

**Figure 4:**
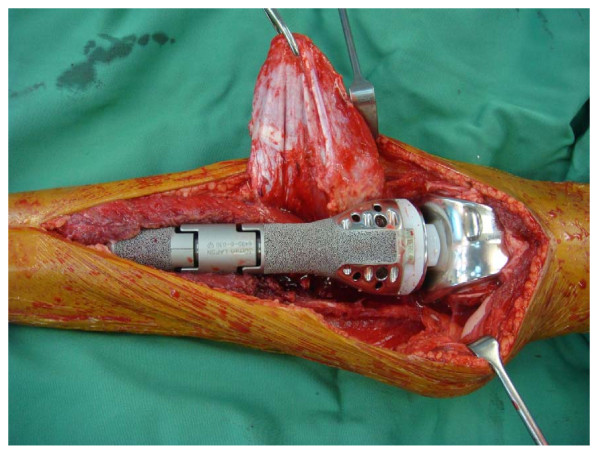
**The endoprosthesis in-situ**.

A standard approach was used and the proximal tibia was resected, followed by proximal tibia endoprosthesis replacement. The resected tumour bone and the endoprosthesis used to replace the defect are shown in Figure 3 and Figure 4. Meticulous attention to haemostasis was of paramount importance. A tourniquet was used during the surgery which was released on and off to secure haemostasis. The patient was operated in Trendelenburg position to minimize blood loss due to high venous pressure when the tourniquet was released. Cell saver technique was not used because of possibility of contamination with malignant cells.

### Acute normovolaemic haemodilution

**Figure 5:**
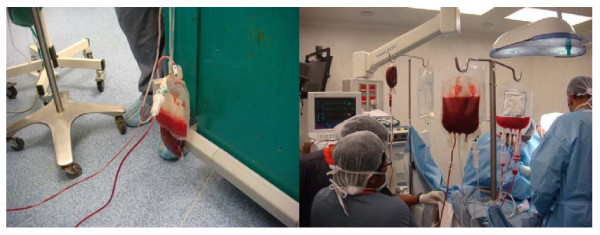
**Autologus blood donation followed by transfusion intraoperatively**.

General anaesthesia with neuromuscular blockade and controlled ventilation was used. A 20 gauge intravenous cannula in the dorsum of the right hand was used to induce anaesthesia. After induction, an 18 gauge cannula was inserted in the right external jugular vein. The Trendelenburg position facilitated drainage of blood. Voluven (hyroxyethyl starch 6%) was infused (in a 1:1 volume ratio for blood extracted) through the right hand cannula to maintain normovolaemia. 400 ml of blood was extracted after which the flow became very sluggish. The blood bag was connected through the second port to the right hand cannula and reinfused without breaking the connection. Another 18 gauge cannula was inserted into the left internal jugular vein and a total of 600 ml of blood was extracted while maintaining normovolaemia. This bag was then inverted and reinfused through the same vein at a slower rate (Figure 5). Total blood loss during surgery was 400 ml which occurred at release of tourniquet and this was replaced introoperatively.

Core temperature as measured with an eosophageal probe was allowed to drop to 33.5°C, which is beneficial to reduce basic metabolic rate, hence, the oxygen requirement. Surgery was uneventful and took about 150 minutes to complete. Postoperatively, the limb was bandaged and elevated to minimize blood loss.

### Postoperative management

The remaining 600 ml of blood was transfused over 6 hours to replace ongoing blood loss as well as to maintain oxygen carrying capacity. Oxygen was administered by face mask at 6 L/min postoperatively. The patient was warmed to normothermia and shivering was prevented. Analgesia was provided by 'patient controlled analgesia' with morphine. All of the above measures reduced oxygen demand and improved oxygen delivery. Her postoperative hemoglobin on the next day was 9.8 g/dL. Meanwhile, the histopathological examination of the resected tumour showed 90% tumour necrosis following neoadjuvant chemotherapy.

**Figure 6:**
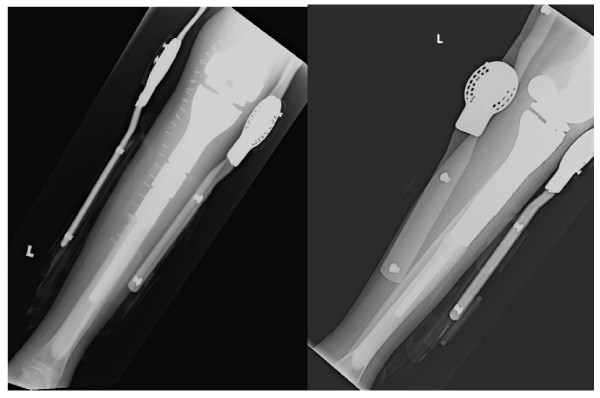
**Postoperative radiographs showing the implant within the bone with an external knee brace**.

Patient was discharged after a week on full weight bearing crutches and hematinics with a hemoglobulin level of 10 g/dL, platelet count of 120 × 10^9^/L and white cell count of 8 × 10^9^/L. Her postoperative radiographs are as shown in figure 6. Adjuvant chemotherapy using the same agents was resumed 3 weeks after the surgery. She completed the remaining 3 cycles of chemotherapy uneventfully.

## Discussion

Jehovah's Witnesses number 7.3 million in the world [2]. Comparatively, this community is very rare in Malaysia with an estimated number of 3,474 or 0.012% of Malaysian population [2]. Nevertheless, medical practitioners in Malaysia will at some point encounter these patients and should be prepared to manage them under various circumstances. Honoring their beliefs can create challenging therapeutic issues especially when it's not in favor of the principle of beneficence and conflicts with best medical practice.

To the medical fraternity, Jehovah's Witnesses are best known for their prohibition on the acceptance of blood transfusion [1]. The blood ban forbids them from accepting transfusion of allogeneic whole blood and its' components which includes red blood cell (RBCs) concentrates, white blood cells, plasma and platelets [1,3]. The management of a case such as osteosarcoma includes the use of high dose chemotherapy and surgery, which entails extensive amount of dissection. Blood loss can be significant and this eventually will require the use of blood product supplements.

Variability exists amongst members of Jehovah's Witnesses about opinions on blood ban. Some patients may accept fractions of blood components or recombinant blood products such as granulocyte colony-stimulating factor (G-CSF), recombinant human erythropoietin and clotting fraction concentrates, whilst others will not [1,4]. Therefore, the patient's preference should be clearly indicated in the medical notes.

During the surgery, normovolaemic haemodilution was utilized, where the autologous blood remains in continuous contact with the patient, with no interruption of the blood circuit [4,5]. This method ensures hemodynamic stability, while maintaining a continuous circuit between the patient and blood bag [5]. Essentially, the technique of acute normovolaemic haemodilution or intraoperative haemodilution involves withdrawing whole blood from the patient into standard collecting blood bags before or shortly after induction of anaesthesia. Normovolaemia is maintained by replacement with crystalloid or colloid solution. The patient's blood can be reinfused intraoperatively and/or postoperatively as was the case in our patient. Haemodilution is an advantage as any blood lost would contain fewer red blood cells per unit volume [6] and the circulating blood volume remains constant.

In addition to that, other strategies to conserve blood such as ensuring effective haemostasis to minimize blood loss and the use of tourniquet during surgery were applied. Tourniquets are normally not used during limb salvage surgery as this makes identifying vessels more difficult, but such a practice can lead to more blood loss. Therefore, for this patient a tourniquet was used for the initial phase of superficial and deep dissection, which was subsequently released when it was time to identify and free the neurovascular structures. Meticulous measures were taken to identify and secure haemostasis at the end of surgery.

Chemotherapy was administered based on Memorial Sloan-Kettering protocol and consisted of doxorubicin, cisplatin and high-dose methotrexate. Preoperative chemotherapy allows immediate treatment of micrometastatic disease, aids in limb preservation and enables assessment of chemotherapy response of the tumour. Optimum survival is normally found in patients with good histologic response of the preoperative chemotherapy (more than 90% tumor necrosis) at the time of surgical resection [7].

During the course of neoadjuvant chemotherapy, the patient's blood counts were monitored both pre and post chemotherapy and haematinics were given from the time of diagnosis to keep her hemoglobin counts high. High-dose recombinant human erythropoietin was also used. It has been shown to significantly increase the haematocrit level with a 50% reduction in the need for blood transfusions [8] and this is acceptable to many Jehovah's Witnesses. The administration of ferrous fumarate, folic acid, recombinant erythropoietin and G-CSF helped to maintain the hemoglobin and white cell counts during the course of chemotherapy and enhanced the preoperative hemoglobin levels to 13.5 g/dL. These measures are important, as a study conducted among patients who declined blood transfusion for religious reasons has shown that morbidity and mortality rates increased dramatically when the hemoglobin concentration decreased below 6 g/dL [9]. Kitchens [10] conducted a review of 16 reports of the surgical outcome of a series Jehovah's Witness patients who were not given blood despite undergoing 1,404 surgical procedures that normally would necessitate transfusion. Lack of blood was the primary cause of death in only 0.6% of patients and a contributor to death in another 0.85% of patients.

Fortunately, the platelet count in our patient was stable during the course of chemotherapy and there were no episodes of bleeding. However, if the need arises, recombinant IL-11 (oprelvekin) which is Food and Drug Administration (FDA) approved can be administered [11]. A systematic review examined the appropriate 'trigger' for platelet transfusion after chemotherapy or stem cell transplantation [12]. The authors found no significant differences in mortality, remission rates, severe bleeding events or RBC transfusion requirements between a transfusion threshold of 10 to 20 × 10^9^/L platelets.

Tenenbaum [13] analyzed the feasibility of oncology treatment in paediatric patients with malignant disease belonging to Jehovah's Witnesses and concluded that such patients can be treated similar to the other patients with a restrictive transfusion policy and broad application of hematopoietic supportive care measures. Also in oncological pediatric patients receiving erythropoietin, a significant reduction in red blood cell and platelet transfusion requirements was shown [14].

While a competent adult patient has an absolute right to refuse medical treatment, the case of adolescents called mature minors, to decline medical treatment is not as straightforward. In some regions, mature minors are given a right for such consent provided that they are deemed to have sufficient understanding and intelligence to make their own decision [15]. Conversely, in other regions, adolescents depend on parental decision-making or that of the courts, if necessary [4,15]. Our patient can be considered a mature minor and consent was obtained both from the parents as well as the patient for the decision to decline blood transfusion. These documentation should absolve all doctors and the hospital from any liabilities should the outcome be adverse as a result of transfusion refusal.

## Conclusion

This case is like any other case of osteosarcoma of proximal tibia with one major difference. This difference lies not in the biological or science aspect, but social believes which has drastic impact on us, the health care providers. This case illustrates how a major disease which required chemotherapy and surgery was carried out successfully in a Jehovah's Witness patient. Building a good rapport with the patient and maintaining effective, honest communication regarding transfusion options without any element of coercion is the cornerstone in the management of these patients. Rather than discriminating Jehovah's Witness patients because of their beliefs, alternative modern medical care acceptable to these patients can be used to support blood volume and haemostatic function, during the course of treatment of serious diseases.

## Competing interests

The authors declare that they have no competing interests.

## Authors' contributions

AD was involved in writing and editing the final manuscript. VAS was the Orthopaedic Oncologist who treated and planned the management of the patient and was involved in critical appraisal of the manuscript. RS the drafted out the initial case report. RR the anesthetist involved in the surgery.

All authors read and approved the final manuscript.

## Authors' Information

**AD**- MBBS, Masters (Path), Consultant Pathologist at Jeffrey Cheah School of Medicine and Health Sciences, Monash University Sunway Campus Malaysia.

**VAJ **- MBBS, FRCS, Masters (Ortho), Consultant Orthopaedic Oncologist and Associate Professor at Department of Orthopaedic Surgery, University Malaya Medical Centre (UMMC)

**RS**- MBBS, Masters (Ortho), Orthopaedic Surgeon at Department of Orthopaedic Surgery, UMMC.

**RR**- MBBS, Masters (Anaes), Consultant Anaesthesiologist at Department of Anaesthesia, UMMC.

## Consent

Written informed consent was obtained from the patient's parents for publication of this case report and accompanying images. A copy of the written consent is available for review by the Editor-in-Chief of this journal.
